# Xanthene Derivatives Increase Glucose Utilization through Activation of LKB1-Dependent AMP-Activated Protein Kinase

**DOI:** 10.1371/journal.pone.0108771

**Published:** 2014-09-24

**Authors:** Yonghoon Kwon, Parkyong Song, Jong Hyuk Yoon, Jaewang Ghim, Dayea Kim, Byungjun Kang, Taehoon G. Lee, Jin-Ah Kim, Joong-Kwon Choi, In Kwon Youn, Hyeon-Kyu Lee, Sung Ho Ryu

**Affiliations:** 1 Department of Life Sciences, Pohang University of Science and Technology (POSTECH), Pohang, Kyungbuk, Republic of Korea; 2 NovaCell Technology Inc., Pohang, Kyungbuk, Republic of Korea; 3 Korea Chemical Bank, Korea Research Institute of Chemical Technology, Daejeon, Republic of Korea; 4 Department of Pharmaceutical Engineering, Pai Chai University, Daejeon, Republic of Korea; CRCHUM-Montreal Diabetes Research Center, Canada

## Abstract

5′ AMP-activated protein kinase (AMPK) is a highly conserved serine-threonine kinase that regulates energy expenditure by activating catabolic metabolism and suppressing anabolic pathways to increase cellular energy levels. Therefore AMPK activators are considered to be drug targets for treatment of metabolic diseases such as diabetes mellitus. To identify novel AMPK activators, we screened xanthene derivatives. We determined that the AMPK activators 9H-xanthene-9-carboxylic acid {2,2,2-trichloro-1-[3-(3-nitro-phenyl)-thioureido]-ethyl}-amide (Xn) and 9H-xanthene-9-carboxylic acid {2,2,2-trichloro-1-[3-(3-cyano-phenyl)-thioureido]-ethyl}-amide (Xc) elevated glucose uptake in L6 myotubes by stimulating translocation of glucose transporter type 4 (GLUT4). Treatment with the chemical AMPK inhibitor compound C and infection with dominant-negative AMPKa2-virus inhibited AMPK phosphorylation and glucose uptake in myotubes induced by either Xn or Xc. Of the two major upstream kinases of AMPK, we found that Xn and Xc showed LKB1 dependency by knockdown of STK11, an ortholog of human LKB1. Single intravenous administration of Xn and Xc to high-fat diet-induced diabetic mice stimulated AMPK phosphorylation of skeletal muscle and improved glucose tolerance. Taken together, these results suggest that Xn and Xc regulate glucose homeostasis through LKB1-dependent AMPK activation and that the compounds are potential candidate drugs for the treatment of type 2 diabetes mellitus.

## Introduction

AMPK is a highly conserved mammalian serine/threonine kinase that can control cellular energy homeostasis by balancing catabolic and anabolic metabolic pathways [1]. AMPK is activated under various physiological conditions, including processes that alter the intracellular AMP/ATP ratio such as exercise, starvation, and hypoxia [2]. AMPK is mainly regulated by two distinct signals: an AMP-dependent pathway mediated by LKB1 and a Ca^2+^-dependent pathway mediated by CamKKb [3]. Once activated, AMPK switches on catabolic processes that stimulate alternative pathways to generate ATP. The enzymes regulated by AMPK are mammalian targets of rapamycin (mTOR), acetyl-CoA carboxylase (ACC), and glycerol phosphate acyltransferase (GPAT), which are key players in protein, fatty acid, and glycerolipid synthesis, respectively [4]–[6]. AMPK activation causes increased glucose uptake through PI3K-independent GLUT4 translocation [7], [8]. Likewise, AMPK is an essential metabolic regulator.

Recently, various AMPK activators such as cytokines, small molecule, and natural compounds have been identified [9]. Metformin, an AMPK activator, is generally used for treatment of type 2 diabetes. In the liver, metformin reduces ACC activity and stimulates fatty acid oxidation through AMPK activation [10]. In skeletal muscle, metformin increases glucose uptake, which results in reduction of blood glucose concentrations through stimulation of AMPK activity [11], [12]. Although it shows beneficial effects on glucose regulation, metformin must be administrated at high doses, and can cause adverse effects such as diarrhea, gastrointestinal symptoms, and lactic acidosis *in vivo* [13]. Therefore, it would be beneficial to identify potent and specific AMPK activators, which could lower the required working concentration for metformin, and thus decrease its adverse effects.

In the present study, we screened potent AMPK activators, identified from a chemical library based on the xanthene structure. In this study, we identified novel AMPK activators, Xn and Xc, which increase glucose uptake *in vitro* through GLUT4 translocation via an LKB1-dependent signaling pathway. Single administration of these molecules activated AMPK in skeletal muscle and ultimately improved blood glucose clearance in high-fat diet-induced diabetic mice. Collectively, our results indicate that these molecules are appropriate candidate drugs for treatment of type 2 diabetes.

## Materials and Methods

### Materials

Metformin (D150959) and *o*-phenylenediamine (P8412) were purchased from Sigma-Aldrich, MO. Xanthene derivatives were synthesized and supplied by Korea Chemical Bank (Daejeon, Korea), and 2- deoxy[^14^C]glucose (ARC0111B) was purchased from American Radiolabeled Chemicals Inc., MO. The AMPK inhibitor, compound c (171260), was purchased from Merck, MA.

### Cell culture and treatment with siRNA

L6 rat skeletal muscle cells were grown in Minimum Essential Medium Alpha (MEM-α, LM008-02, Welgene, Korea) containing 10% fetal bovine serum (FBS), 50 units of penicillin/mL and 50 µg of streptomycin/mL at 37°C, 5% CO_2_ in a humidified incubator. Differentiation of L6 rat skeletal muscle cells was induced by incubation in 2% FBS containing MEM-α for 1 week. STK11 #1 siRNA (5′-CUCCAUCCGACAGAUUAGA-3′) and STK11 #2 siRNA (5′-CGACAGAUUAGACAGCACA-3′) were custom synthesized from bioneer (Daejeon, Korea). As a control siRNA, we used pre-designed luciferase siRNA purchased from bioneer (SS1013, Daejeon, Korea). siRNA were used at 100 nM to transfect L6 myotubes using lipofectamin LTX (1533810, Invitrogen, CA) as manufacturer's instruction at 6th day after differentiation of L6 myotubes.

### Western blotting

To prepare total cell lysate, plated cells were washed with cold PBS and then lysed with cold lysis buffer containing 40 mM HEPES, 120 mM NaCl, 1 mM EDTA, 10 mM pyrophosphate, 10 mM glycerophosphate, 50 mM NaF, 1.5 mM Na_3_VO_4_, 1 mM PMSF, 5 mM MgCl_2_, 0.5% Triton X-100, and protease inhibitor mixture. Following SDS-PAGE and transfer to a nitrocellulose membrane, each molecular size of nitrocellulose membrane was incubated with primary antibody (1∶1000) overnight at 4°C using the following antibodies: anti-AMPK (07-181, Upstate, NY), anti-phospho-AMPK*a* thr-172 (4188S), anti-ACC (3676S), anti-LKB1 (3047S, Cell Signaling Technology, MA), and anti-phospho-ACC ser 79 (07-303, Millipore, MA).

### 2-Deoxy [^14^C] glucose uptake assay

The assay for 2-deoxy [^14^C] glucose uptake was performed as previously reported [14]. The indicated agents were administered to myotubes for 1 h following 3 h of incubation in MEM-α without FBS. Cells were pre-incubated for 30 min prior to treatment with AMPK activators. Cells were washed twice with Krebs-Henseleit buffer and glucose levels were measured with 0.1 mCi/mL 2-Deoxy [^14^C] glucose at room temperature for 10 min.

### Myc-GLUT4 translocation assay

The antibody-based quantification of the plasma membrane located GLUT4 was determined by *o*-phenylenediamine (OPD) and immunocytochemistry as previously described [15]. The cell was treated with indicated agents for 1 h following 3 h of incubation in MEM-α without FBS. Cells were washed twice with PBS after treatment and then incubated with anti-Myc antibody (05-724, Millipore Corp, MA) to label myc-GLUT4 expressing L6 myotubes. After incubation with the primary antibody, peroxidase-labeled anti-mouse IgG secondary antibody (074-1806, KPL, MD) for OPD assay or alexa488 labeled anti-mouse IgG secondary antibody (A11001, Invitrogen, CA) for immunocytochemistry were added. The immunocytochemistry sample was imaged by confocal microscopy (LSM700, Zeiss).

### AMP and ATP measurement

L6 myotubes were lysed after treatment with the indicated agents by trichloroacetic acid. The AMP and ATP levels were determined by high-performance liquid chromatography after nucleotide extraction as previously describe [16].

### Animal experiments

All animal experimental procedures were approved by the Pohang University of Science and Technology (POSTECH) Animal Use and Care Committee. Male C57Bl/6J mice 4–5 weeks old were kept in a 12 h light/dark cycle with free access to water. To produce insulin-resistance mice, mice were fed a high-fat diet with 60% kcal fat for 10 weeks. To confirm insulin resistance, we measured the body weight and fasting glucose levels of the mice fed a high-fat diet prior to the experiments. Xanthene derivatives was dissolved in 10% DMSO in Tween 80/saline. 30 min after intravenous injection of the indicated reagents, we measured blood glucose levels using a glucometer (Accu-Check Active; Roche Diagnostics) from blood taken from the tail vein at the indicated time. To test glucose tolerance, mice were fasted overnight followed by the administration of an intraperitoneal dose of 1 g/kg of glucose (G7021, Sigma-Aldrich, MO) 30 min after injection of the indicated reagents. Blood insulin level was measured by Mouse Insulin ELISA (80-INSMS-E01, ALPCO, NH) according to manufacturer's instruction. Plasma sample were collected by orbital eye bleeding after one-week administration of indicated reagents.

### Statistical analysis

All data are expressed as mean ± SE. Statistical analyses were performed using a one-way ANOVA. Tukey's *t*-test was used for multiple comparisons. Differences with a *P*- value of <0.05 were considered statistically significant.

## Results

### Xn and Xc activate AMPK in L6 myotubes

**Figure 1:**
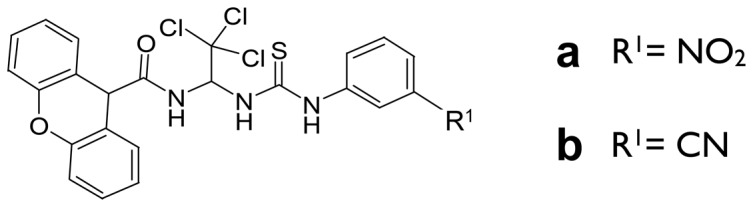
Structure of Xn and Xc. The chemical structure of 9H-xanthene-9-carboxylic acid {2,2,2-trichloro-1-[3-(3-R^1^-phenyl)-thioureido]-ethyl}-amide: (a) 9H-xanthene-9-carboxylic acid {2,2,2-trichloro-1-[3-(3-nitro-phenyl)-thioureido]-ethyl}-amide (Xn) and (b) 9H-xanthene-9-carboxylic acid {2,2,2-trichloro-1-[3-(3-cyano-phenyl)-thioureido]-ethyl}-amide (Xc).

**Figure 2:**
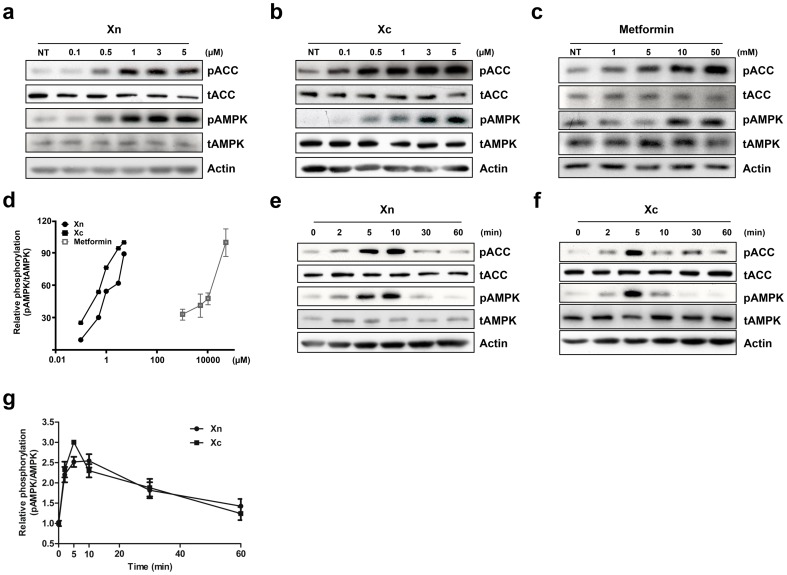
Xn and Xc activate AMPK in L6 myotubes. The xanthene derivatives Xn (a), Xc (b) and metformin (c) increased phosphorylation of AMPK*a*2 thr-172 and ACC ser-79 in a dose-dependent manner. The indicated concentration was administered for 5 min for Xn and Xc, and 2 h for metformin. (d) Concentration–effect curves of three AMPK activators. Each curve is based on quantification of individual experiments allowing comparison of the effective concentration between metformin and xanthene derivatives. The time-dependent phosphorylation trend of AMPK *a*2 thr-172 following treatment with the xanthene derivatives Xn (e) and Xc (f). A 5 µM concentration of xanthene derivatives was administered to L6 myotubes at the indicated time point. The numbers indicate min following administration. (g) The graph shows the time-dependent trend in AMPK-phosphorylation induced by Xn and Xc. Western blot data represent one of three independent experiments. Values shown in graphs are means ± SE from three independent experiments.

To discover novel AMPK activators that may be potential metabolic candidate drugs, we performed broad molecule screening based on xanthene backbone. We identified two compounds derived from 9H-xanthene-9-carboxylic acid {2,2,2-trichloro-1-[3-(3-R^1^-phenyl)-thioureido]-ethyl}-amide (Fig. 1). Each compound contains either a nitro (Fig. 1-a) or a cyano R^1^ (Fig. 1-b) group, and they are referred to as Xn and Xc. To clarify the biochemical properties of Xn and Xc, we administered each compound in a dose- and time-dependent manner to L6 myotubes. Both compounds induced AMPK phosphorylation at a 1 µM concentration (Fig. 2a and b). We compared the EC_50_ values of both compounds with metformin that induces AMPK phosphorylation at a 10 mM concentration (Fig. 2c). Xn and Xc showed an EC_50_ value of approximately 1.5 µM, much lower than metformin; metformin phosphorylates AMPK at 10 mM (Fig. 2d). Activation occurred at 5 min after administration of the compounds (Fig. 2e and f). AMPK phosphorylation was strongly maintained from 2 to 10 min and gradually decreased after 10 min (Fig. 2g). In addition, similar patterns of ACC phosphorylation were observed under the same conditions. These data indicate that the xanthene-derived compounds Xn and Xc activate the AMPK-ACC pathway in differentiated L6 myotubes cells at a significantly lower concentration than metformin.

### Xn and Xc increase glucose uptake by stimulation of GLUT4 translocation

**Figure 3:**
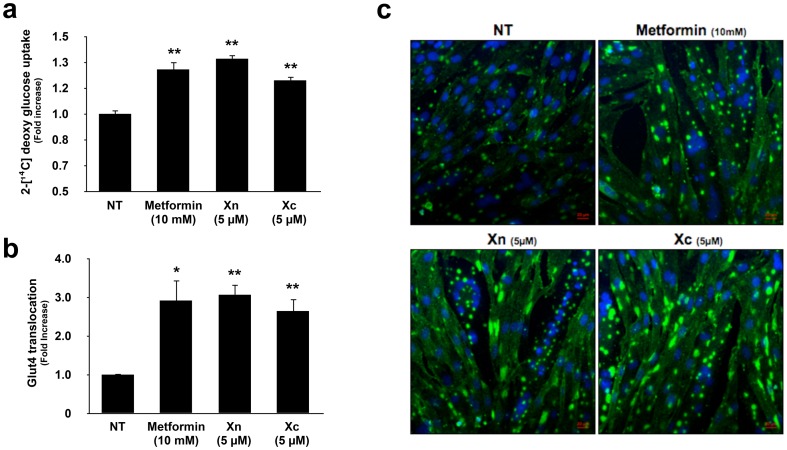
Xn and Xc increase glucose uptake by stimulating GLUT4 translocation. (a) L6 myotubes were incubated in glucose-free Krebs-Henseleit buffer before measurement of glucose levels for 1 h and then incubated with the indicated agents for 1 h. 2-Deoxy [^14^C] glucose uptake was measured as described in the Materials and Methods. (b, c) To determine GLUT4 translocation to plasma membrane, Xn, Xc, and metformin was administered to L6 myotubes for 1 h at the indicated concentrations, respectively. The *o*-phenylenediamine assay (b) and immunocytochemistry (c) was performed as described in the Materials and Methods. Values show the mean ± SE. of three independent experiments performed in triplicate. *, *P*<0.05 and **, *P*<0.01 versus non-treated control (NT).

In order to evaluate the roles of Xn and Xc in glucose utilization, we investigated the level of glucose uptake in L6 myotubes. We observed that both compounds increased glucose uptake at a concentration of 5 µM, which is similar to the concentration required for phosphorylation of AMPK (Fig. 3a). To clarify the mechanism of glucose uptake, we measured the level of cell-surface GLUT4. Plasma membrane-localized GLUT4 was detected by an OPD-based biochemical assay and immunocytochemistry. The level of GLUT4 translocation to the plasma membrane increased after treatment with Xn and Xc under the same dose and time conditions used in the glucose uptake assay (Fig. 3b and c). Treatment with 10 mM metformin displayed relatively similar effects on glucose uptake and GLUT4 translocation to treatment with Xn or Xc. Collectively, our findings suggest that Xn and Xc are potent AMPK activators that increase glucose uptake in L6 myotubes via GLUT4 translocation.

### Inhibition of AMPK eliminates Xn- and Xc-induced glucose uptake

**Figure 4:**
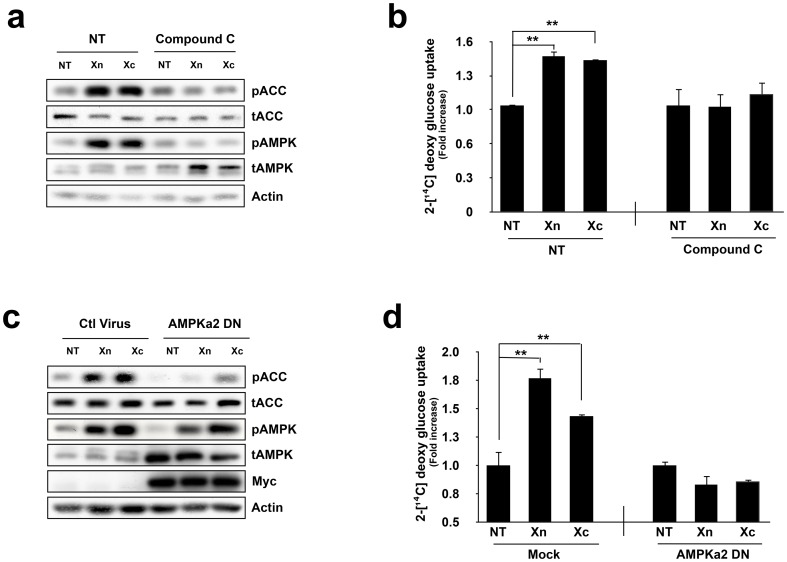
Inhibition of AMPK eliminates Xn- and Xc-induced glucose uptake. (a) L6 myotubes were pre-incubated with the AMPK chemical inhibitor, compound c, for 30 min and then treated with the indicated agents for 5 min at a 5 µM concentration. Data represent one of three independent experiments. (b) L6 myotubes were equilibrated in glucose-free Krebs-Henseleit buffer for 1 h and then incubated with the indicated agents, with or without pre-incubation with compound c for 30 min. 2-Deoxy [^14^C] glucose uptake was measured, in the Materials and Methods. (c) L6 myotubes were infected with a mock or dominant negative AMPK *a*2 adenovirus for 48 h. After infection, the indicated agents were administered for 5 min at a 5 µM concentration. Infection with the adenovirus was confirmed by detection of an anti-Myc antibody. (d) Mock and dominant negative AMPK *a*2 adenovirus infected L6 myotubes was equilibrated in glucose-free Krebs-Henseleit buffer for 1 h and then incubated with the indicated agents for 1 h. 2-Deoxy [^14^C] glucose uptake was measured in the Materials and Methods. Western blot data represent one of three independent experiments. Values in graphs are mean ± SE. of three independent experiments performed in triplicate. *, *P*<0.05 and **, *P*<0.01 versus non-treated control.

To verify the specificity of the AMPK signal pathway in the enhancement of glucose uptake induced by treatment with Xn and Xc, we used two different methods: (1) treatment with a chemical AMPK-specific inhibitor, compound C, and (2) infection with a dominant negative AMPK*a2* virus in which Asp^157^ was replaced with alanine, an abundant isoform of the AMPK*a* subunit found in skeletal muscle. Following pre-incubation with compound C, we observed that Xn- and Xc-induced phosphorylation of AMPK and ACC were significantly decreased (Fig. 4a). Next, we verified glucose uptake by L6 myotubes. The increased level of glucose uptake induced by treatment with Xn and Xc was significantly eliminated following pre-treatment with compound C (Fig. 4b). Moreover, infection with the dominant negative AMPK*a2* virus decreased Xn- and Xc-induced activation of signaling downstream of AMPK (Fig. 4c). This result was consistent with glucose uptake levels (Fig. 4d). Collectively, Xn and Xc increased glucose uptake in L6 myotubes via the AMPK signaling pathway.

### Xn and Xc activate AMPK via the LKB1 signaling pathway

**Figure 5:**
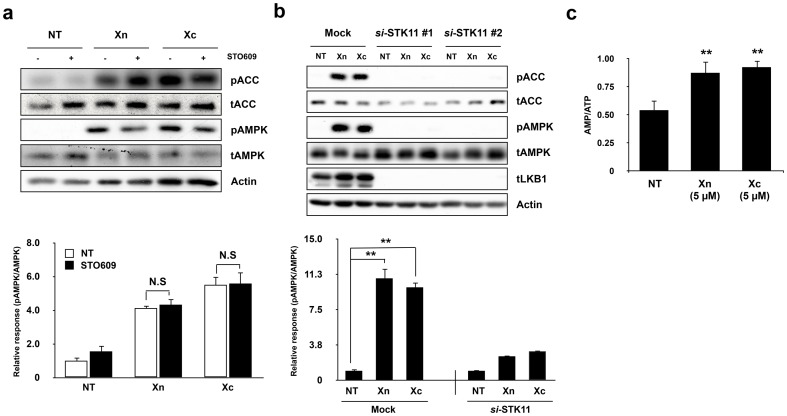
Xn and Xc activate AMPK via the LKB1 signaling pathway. (a) Indicated agents were treated for 5 min and 5 µM concentration with or without 30 min pre-incubation of 2.5 µM concentration of STO609 in L6 myotubes. (b) L6 myotubes was transfected by mock or two different LKB1 siRNA for 48 h. After infection, indicated agents were treated for 5 min and 5 µM concentration. The graphs show quantification of western blot based on densitometric analysis. (c) Indicated agents were treated for 5 min and 5 µM concentration to L6 myotubes. Celluar AMP/ATP ratio was measured by high-performance liquid chromatography as described in Materials and Methods. Western blot data represent one of three independent experiments. Values are mean ± SE. of three independent experiments. N.S means not significant. *, *P*<0.05 and **, *P*<0.01 versus non-treated controls.

To verify the mechanism of AMPK activation by Xn and Xc, we tested the involvement of major upstream regulators of AMPK, including CaMKK and LKB1. First, we pre-incubated the cells with a chemical inhibitor of CaMKK, STO609, to verify the possibility that AMPK phosphorylation via the CaMKK pathway. Inhibition of CaMKK had no effect on the phosphorylation of AMPK (Fig. 5a). To confirm that Xn- and Xc-induced AMPK activation is dependent on LKB1, we silenced STK11, the rat ortholog of human LKB1, using two different siRNAs. Knockdown of STK11 in L6 myotubes eliminated Xn- and Xc-induced phosphorylation of AMPK and ACC (Fig. 5b). Because LKB1-dependent AMPK activation is caused by an increase in cellular AMP/ATP ratio, we measured the change in AMP/ATP ratio in L6 myotubes after treatment with the indicated reagents. As a result, Xn and Xc increased the cellular AMP/ATP ratio (Fig. 5c). This shows that Xn and Xc activate AMPK via an LKB1-dependent pathway.

### Xn and Xc increase AMPK activity and glucose utilization in high-fat diet-induced diabetic mice

**Figure 6:**
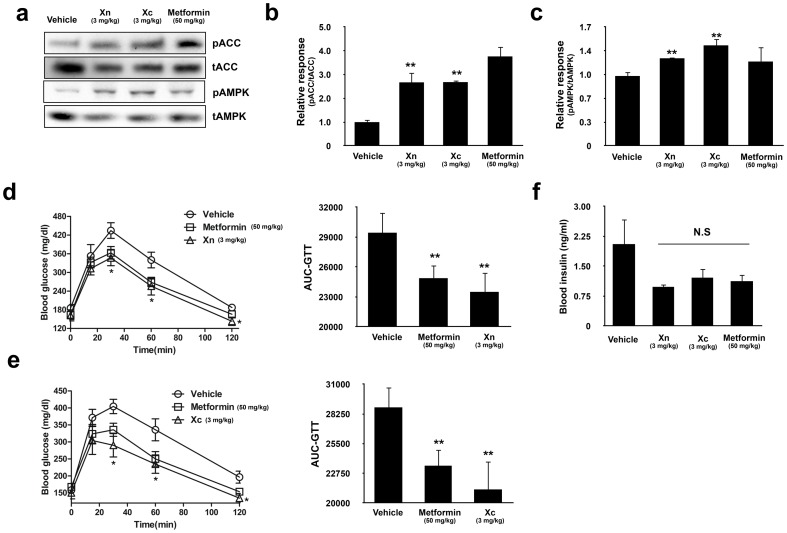
Xn and Xc increase AMPK activity and glucose utilization in high-fat diet-induced diabetic mice. (a) Phosphorylation of AMPK and ACC in the skeletal muscle of high-fat diet-induced diabetic mice model after a single intravenous injection of the indicated concentration of agents. Densitometric analysis of phosphorylation of (b) ACC and (c) AMPK in the skeletal muscle of 4 different individual high-fat diet-induced diabetic mice. Blood glucose levels were measured after intraperitoneal glucose injection (1 g/kg) following a single intravenous administration of (d) Xn and (e) Xc with metformin, at the indicated concentrations to high-fat diet-induced diabetic mice. The graph on the right shows the area under the curve (AUC). (f) Plasma insulin level was measured by orbital eye bleeding after 1 week administration of indicated agents. Results are the mean ± SE of six mice per group (n = 6). One-way analyses of variance and Tukey's multiple comparisons tests were performed to determine the significance of the results of the glucose tolerance tests. *, *P*<0.05 and **, *P*<0.01 versus vehicle treatment.

Next, we validated the ability of Xn and Xc to upregulate the AMPK signaling pathway *in vivo*. We injected a 3 mg/kg dose of Xn and Xc intravenously into high-fat diet-induced diabetic mice, because it was sufficient for the significant glucose uptake without any apparent side effects. The administration of Xn and Xc significantly increased phosphorylation of AMPK and ACC in the skeletal muscle of the mice, and these increases were comparable to the increase produced by a 50 mg/kg dose of metformin (Fig. 6a–c). We tested the ability of Xn and Xc to enhance glucose tolerance in high-fat diet-induced diabetic mice using a glucose tolerance test (GTT). We observed a clear improvement in glucose tolerance from 30 to 120 min following a glucose injection through measuring blood glucose values at each time point. The area under the curve for the GTT was also significantly decreased in the treatment groups (Fig. 6d, e). The observed improvements were equivalent to those observed following a relatively high dose (50 mg/kg) of metformin. Furthermore we checked blood insulin level to confirm that improve of glucose clearance after administration of Xn and Xc was not related with acute secretion of insulin. As a result, blood insulin level was not affected by treatment of Xn and Xc (Fig. 6f). Collectively, we confirmed *in vivo* that a 3 mg/kg dose of Xn or Xc can not only upregulate the AMPK signaling pathway but also increase glucose clearance without acute secretion of insulin, similar to a 50 mg/kg dose of metformin.

## Discussion

We identified two novel AMPK activators, Xn and Xc, which improve glucose homeostasis when administered at a very low concentration. We confirmed that increased GLUT4 translocation induced glucose uptake via LKB1-dependent AMPK activation *in vitro* and improved glucose tolerance by AMPK activation in skeletal muscle *in vivo*, these compounds may be attractive candidate drugs for treatment of type 2 diabetes.

Blood glucose utilization is mainly regulated by skeletal muscle, which is responsible for ∼75% of the glucose uptake in the whole body [17]. In particular, skeletal muscle AMPK plays a role in increasing GLUT4 translocation, which contributes to glucose uptake [18]. To determine whether Xn and Xc activate AMPK and increase glucose uptake *in vivo*, we performed a single intravenous administration of Xn and Xc in high-fat diet-induced diabetic mice. A single injection of 3 mg/kg of Xn and Xc increased phosphorylation of AMPK and ACC in skeletal muscle. Glucose tolerance was also increased by administration of Xn and Xc. Those effects were not affected by implication of insulin-dependent signaling molecules such as AKT (data not shown). Because present study was performed in high-fat diet-induced diabetic mice, a commonly used model for diabetes, our results suggest the therapeutic potential of Xn and Xc for AMPK activation in skeletal muscle and whole-body glucose utilization. In addition to the single-administration effects of Xn and Xc in glucose utilization, it is important to study their long-term administration effects in a high fat diet-induced diabetic model. Chronic activation of AMPK could be responsible for increased glycolysis by stimulating glucose uptake [19] and hexokinase activity [20], [21]. Additionally AMPK activators block glycogen synthase to decrease glycogen content [22]. In fat metabolism, activation of AMPK inhibits ACC that can stimulate fat oxidation [23]. In the future, Xn and Xc could be chronically administrated to improve the effects of other AMPK activators.

Compared to other types of anti-diabetic drugs such as metformin, the dosages of Xn and Xc are very low so there is a decreased possibility of over dosage adverse effects, such as lactic acidosis, diarrhea, and gastrointestinal side effects [24]–[26]. In this study we observed that Xn and Xc are much more potent than metformin. In particular, the EC_50_ value of Xn and Xc is approximately 6000-fold less than metformin *in vitro*. Compared to previously reported values for other AMPK activators, EC_50_ values for Xn and Xc were 100-fold and 20-fold less those for resveratrol and TZD, respectively [27], [28]. Additionally, we verified that administration of 3 mg/kg of Xn and Xc produced similar improvements in glucose tolerance to administration of 50 mg/kg of metformin *in vivo*; thus, the experimental dosages of Xn and Xc were an order of magnitude lower than metformin. This dosage used here was also 300, 80, and 10-fold lower than doses of AICAR, salicylate, and A-769662 [29], [30].

LKB1 and CamKK are both upstream kinases of AMPK. LKB1 has been reported to be the primary regulator of AMPK activation in metabolic organs, whereas hypothalamic neurons [31], T cells [32], and endothelial cells [33] are regulated by CamKK. Several AMPK activators such as metformin and TZDs that target mitochondrial complex 1 have been reported to induce changes in the AMP/ATP ratio, which is related to LKB1-dependent AMPK activation [34]. On the other hands, inhibition of complex 1 has been reported to enhance mitochondrial ROS production [35], [36]. ROS have been shown to activate AMPK through both LKB1- and CamKK-dependent pathways [37], [38]. Thus, AMPK activators that target mitochondrial complex 1 have the potential to induce ROS-dependent, Ca^2+^-related, CamKK-dependent AMPK activation [34]. In contrast, Xn and Xc clearly show LKB1-dependent AMPK activation. We confirmed that knockdown of LKB1 eliminated AMPK signaling, and treatment with the Ca^2+^ signaling blocker, STO609, produced no effect on Xn- and Xc-induced AMPK activation. Therefore, we suggest that Xn and Xc are LKB1-specific AMPK activators.

We screened the chemical library for structures containing xanthene, because xanthene forms the chemical backbone structure of mangiferin, which is a traditional drug, used to treat diabetes in Southeast Asia. However, its molecular mechanism has not been well studied and the appropriate dosage has yet to be determined [39], [40]. However, the xanthene backbone structure we used in our screening step has less effect on AMPK activation than Xn and Xc (data not shown). Remarkably, Xn and Xc share urea and phenyl moieties, and these unique structures may contribute in a dose-dependent manner to the increased activation of AMPK. Thus synthesis of several series of compounds will be required to confirm the pharmacophore of Xn and Xc.
